# Efficacy of Anti-Interleukin-2 Receptor Antibody in Reducing the Incidence of Acute Rejection After Renal Transplantation

**DOI:** 10.5812/numonthly.7279

**Published:** 2012-09-24

**Authors:** Alexandre Braga Liborio, Tacyano Tavares Leite

**Affiliations:** 1 University of Fortaleza – UNIFOR, Fortaleza, Brazil; 2 Hospital Geral de Fortaleza, Fortaleza, Brazil

**Keywords:** Kidney transplantation, Therapeutics, Rejection

Dear Editor,

Acute rejection (AR) in kidney transplant recipients is a major risk factor for chronic allograft failure and reduction in early and long term mean graft survival. New immunosuppressant agents have been developed to reduce the incidence of these episodes (1). Immunobiological drugs such as monoclonal anti-interleukin-2 receptor antibodies have shown promise in this direction; they have a more restrict immunosuppressive effect, are not related to interleukin release syndrome and are related to lower rates of CMV infection than polyclonal antibodies (2).

In his trial Saghafi et al. (3) showed a reduction of Biopsy-Proven Acute Rejection (BPAR) episodes in non-related living donor kidney transplant recipients performing anti-interleukin-2 receptor, daclizumab, as an induction drug in addition to standard therapy with cyclosporine, mycophenolate and prednisone. The induction group had a statistically significant (20.8%) reduction in rejection episodes compared to control group using only standard therapy after a six month follow up (3). 

Resembling data had already been shown in previous studies, Vincent et al. showed similar reduction in incidence of BPAR in low immunologic risk deceased donor kidney recipients and the same rate of bacterial infections and/or viral infections, including CMV, malignances and adverse event as placebo group (4). Another study showed a reduction in the incidence of BPAR in deceased and living kidney recipients of about 14 % in the group that underwent induction therapy with daclizumab with no increase in infections and adverse events (1).

Saghafi et al. in accordance with other authors showed daclizumab as an effective induction treatment option for low immunologic risk living donor kidney recipients. However, missing data about safety drug profile such as incidence of infection, adverse events, CMV infection, and early and late cyclosporine trough levels are not reported in the text.

There are several studies confirming that induction therapy with either monoclonal or polyclonal antibodies in addition to standard therapy reduces the incidence of acute rejection but none of these studies have shown statistically significant improving in long term graft survival. New trials such as ELITE-symphony (5) study performing inteleukin-2 receptor antibody as a calcineurin inhibitor sparing agent showed promising results when comparing classical treatment protocol with standard cyclosporine dose, mycophenolate and corticosteroids with daclizumab, corticosteroids and mycophenolate and low dose cyclosporine or low dose tacrolimus or low dose sirolimus. This study found better outcomes in term of lower incidence of acute rejection, adverse events, infections and a better allograft survival rates in daclizumab and low dose tacrolimus group (5).

Improving long-term outcomes in renal transplantation is still a field of challenge and numbness but the use of induction agents in adjunction with other drugs for reducing their side effects is a promising strategy for better graft and patient survival. Perhaps, most important induction therapy importance is in minimizing exposure to other immunosuppressive therapy and its side effects and this topic deserves research.
